# Intravitreal Anti-Vascular Endothelial Growth Factor Agents for the Treatment of Diabetic Retinopathy: A Review of the Literature

**DOI:** 10.3390/pharmaceutics13081137

**Published:** 2021-07-26

**Authors:** Irini Chatziralli, Anat Loewenstein

**Affiliations:** 1 2nd Department of Ophthalmology, National and Kapodistrian University of Athens, 12462 Athens, Greece; eirchat@yahoo.gr; 2 Division of Ophthalmology, Tel-Aviv Sourasky Medical Center, Tel Aviv-Yafo 6423906, Israel; 3 Sackler Faculty of Medicine, Tel-Aviv University, Tel Aviv-Yafo 6997801, Israel

**Keywords:** diabetic retinopathy, ranibizumab, aflibercept, trials, proliferative, ischemia, anti-VEGF agents

## Abstract

**Background:** Diabetic retinopathy (DR) is the leading cause of blindness in the working-age population. The purpose of this review is to gather the existing literature regarding the use of the approved anti-vascular endothelial growth (anti-VEGF) agents in the treatment of DR. **Methods:** A comprehensive literature review in PubMed engine search was performed for articles written in English language up to 1 July 2021, using the keywords “diabetic retinopathy”, “ranibizumab”, “aflibercept”, and “anti-VEGF”. Emphasis was given on pivotal trials and recent robust studies. **Results:** Intravitreal anti-VEGF agents have been found to significantly improve visual acuity and reduce retinal thickness in patients with diabetic macular edema (DME) in a long-term follow-up ranging from 1 to 5 years and are considered the standard-of-care in such patients. Regarding DR, intravitreal anti-VEGF agents provided ≥2-step improvement in DR severity on color fundus photography in about 30–35% of patients with NPDR at baseline, in the majority of clinical trials originally designed to evaluate the efficacy of intravitreal anti-VEGF agents in patients with DME. Protocol S and CLARITY study have firstly reported that intravitreal anti-VEGF agents are non-inferior to panretinal photocoagulation (PRP) in patients with proliferative DR (PDR). However, the use of new imaging modalities, such as optical coherence tomography-angiography and wide-field fluorescein angiography, reveals conflicting results about the impact of anti-VEGF agents on the regression of retinal non-perfusion in patients with DR. Furthermore, one should consider the high “loss to follow-up” rate and its devastating consequences especially in patients with PDR, when deciding to treat the latter with intravitreal anti-VEGF agents alone compared to PRP. In patients with PDR, combination of treatment of intravitreal anti-VEGF agents and PRP has been also supported. Moreover, in the specific case of vitreous hemorrhage or tractional retinal detachment as complications of PDR, intravitreal anti-VEGF agents have been found to be beneficial as an adjunct to pars plana vitrectomy (PPV), most commonly given 3–7 days before PPV, offering reduction in the recurrence of vitreous hemorrhage. **Conclusions:** There is no general consensus regarding the use of intravitreal anti-VEGF agents in patients with DR. Although anti-VEGF agents are the gold standard in the treatment of DME and seem to improve DR severity, challenges in their use exist and should be taken into account in the decision of treatment, based on an individualized approach.

## 1. Introduction

**Figure 1:**
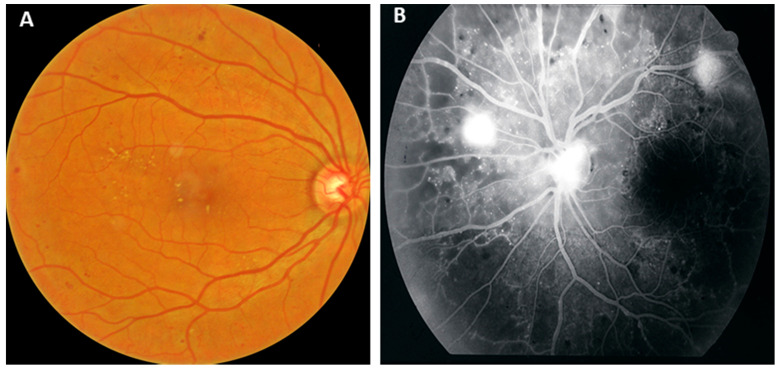
(**A**) Color fundus photograph of a patient with non-proliferative diabetic retinopathy (moderate-to-severe). (**B**) Fluorescein angiography of a patient with proliferative diabetic retinopathy, where neovascularization on the disc or elsewhere are depicted as hyperreflective leakage points. Note also non-perfusion areas as hypofluorescence especially in the periphery.

Diabetes mellitus is a global growing epidemic, affecting more than 400 million people worldwide, a number estimated to reach over 640 million by 2040 [1,2,3]. Diabetic retinopathy (DR) is the most common microvascular complication of diabetes and is considered the leading cause of blindness in patients aged between 20 and 74 years [4,5,6]. It is classified as non-proliferative (NPDR) or proliferative DR (PDR) based on the presence of neovascularization on the optic disc (NVD) or elsewhere (NVE) (Figure 1), while PDR may lead to devastating complications, such as vitreous hemorrhage (VH) or tractional retinal detachment (TRD) [5]. Of note, in the USA approximately 30% of adult patients with diabetes are found to have some form of DR and almost 3% diabetic macular edema (DME), which can occur at any stage of DR [7]. According to a recent meta-analysis, in 2020 the number of adults worldwide with DR was estimated to be 103 million and is projected to increase to 160.5 million by 2045 [8].

In the pathogenesis of DR, including DME, chronic hyperglycemia promotes biochemical alterations and consequent structural changes in the retinal blood vessels’ wall. The latter include pericyte loss and endothelial cell damage leading to disruption of the blood–retina–barrier and consequent vascular hyperpermeability, while thickening of retinal capillaris’ basement membrane ends up to vessel closure, capillary drop-out, and non-perfusion [3,9,10,11]. In both pathways, namely, vascular hyperpermeability and ischemia, vascular endothelial growth factor (VEGF) upregulation has been found to be the most prominent factor [12,13,14]. Specifically, VEGF-A is a key signaling glycoprotein, triggering endothelial cell proliferation, cell migration, vascular leakage and angiogenesis in oxygen-deprived tissues, being also the most potent angiogenic molecule among the other VEGF family members [15].

Before the availability of intravitreal anti-VEGF agents, the treatment of DR was limited to laser photocoagulation and control of systemic factors [3,5]. Anti-VEGF agents are today the standard-of-care in the treatment of DME with large randomized clinical trials to show their efficacy and safety [16,17,18]. Of note, two anti-VEGF agents have been approved by the United States Food and Drug Administration (FDA) for ocular use, ranibizumab and aflibercept. However, besides the treatment of DME, note that in post hoc analyses of RISE (“A Study of Ranibizumab Injection in Subjects With Clinically Significant Macular Edema With Center Involvement Secondary to Diabetes Mellitus”) and RIDE (“A Study of Ranibizumab Injection in Subjects With Clinically Significant Macular Edema With Center Involvement Secondary to Diabetes Mellitus”), as well as VIVID (“Intravitreal Aflibercept Injection in Vision Impairment Due to DME”) and VISTA (“Study of Intravitreal Aflibercept Injection in Patients With Diabetic Macular Edema”) studies, the two approved intravitreal anti-VEGF agents, ranibizumab and aflibercept respectively, have been found to improve DR severity over time in patients with DME and moderately severe or severe NDPR [19,20]. It has to be mentioned that besides ranibizumab and aflibercept, another anti-VEGF agent—brolucizumab—has been recently approved for ocular indications, i.e., age-related macular degeneration, while studies regarding DME are still ongoing.

In cases of PDR, panretinal photocoagulation (PRP) has been considered the gold standard for its treatment for many years, as the pivotal Diabetic Retinopathy Study has shown that PRP reduces the risk of severe visual loss by 50% in patients with high-risk PDR [21]. Nevertheless, PRP may have complications, such as permanent peripheral visual field loss, epiretinal membrane development and worsening of DME, while about 5% of eyes with PDR exhibit severe vision loss despite timely PRP [21]. New evidence shows that both PRP and intravitreal anti-VEGF agents could be considered as viable treatment options either alone or in combination [22,23,24,25,26,27].

On the other hand, intravitreal dexamethasone implant has been found to improve retinal perfusion in patients with treatment naïve DR and DME, probably due to its positive effect in leukostasis [28]. In addition, Iglicki et al. in a retrospective multicenter study have reported that intravitreal dexamethasone implant has the potential to not only delay the progression of DR, including PDR development, but may also improve DR severity over 24 months [29].

Based on the above, the purpose of this review is to scrutinize the existing literature regarding the use of the approved intravitreal anti-VEGF agents, namely ranibizumab and aflibercept, in the treatment of DR, giving special emphasis on the results of robust, randomized studies.

## 2. Methods

A comprehensive literature review in PubMed engine search was performed, using the algorithm ((diabetic retinopathy) AND (aflibercept OR ranibizumab OR Eylea OR Lucentis OR anti-VEGF)) for articles in English language up to 1 July 2021. All abstracts derived using this algorithm were reviewed, while the references’ lists of the selected papers were examined to find additional articles. 

## 3. Results

Table 1 summarizes the main findings of the most important and robust randomized studies regarding the use of anti-VEGF agents for the treatment of diabetic retinopathy.

### 3.1. Ranibizumab in the Treatment of Diabetic Retinopathy

Ranibizumab is a recombinant, humanized, monoclonal antibody fragment (Fab) with a molecular weight of 48 kDaltons. It has a high affinity to VEGF-A, inhibiting its action and blocking its stimulation of VEGFR1 and VEGFR2 receptors [37]. Specifically, VEGF-A is a key signaling glycoprotein, triggering endothelial cell proliferation, cell migration, vascular leakage, and angiogenesis in oxygen-deprived tissues, while it exerts its biological effects after binding to two tyrosine kinase receptors (VEGFR1 and VEGFR2) located on cell surfaces [15,38,39]. Ranibizumab binds to both VEGFR1 and VEGFR2, preventing the interaction of VEGF-A with these receptors [37]. Interestingly, ranibizumab does not include the Fc antibody region, which is associated with immune system activation [37].

Intravitreal ranibizumab (Lucentis^®^, Novartis, Basel, Switzerland) has been approved by the FDA for all forms of DR and recently by European Medicines Agency (EMA) for PDR, based on robust clinical trials [40,41]. The pivotal phase III clinical trials RISE and RIDE included patients with DME treated with ranibizumab and offered the first opportunity to evaluate its effect on DR [19,42,43]. In these studies, patients with DME were randomized to receive monthly intravitreal injections of 0.3 mg or 0.5 mg ranibizumab or sham for 24 months, while during months 24–36, patients in the sham group allowed to cross over to active treatment with 0.5 mg ranibizumab [42,43]. A post hoc analysis of the RISE and RIDE trials evaluated the DR outcomes through month 36 by baseline DR severity, showing that 35.7–38.5% of ranibizumab-injected eyes presented an improvement in their retinopathy compared to 4–7% in the sham group [19]. Note that the greatest benefits in DR improvement occurred in patients with baseline moderately severe to severe NPDR (DR severity levels 47/53) [19]. In addition, in patients with baseline severe NPDR, ranibizumab reduced the probability of experiencing a new proliferative event at month 36 by 3 times compared with sham treatment (12.4% and 11.9% vs. 35.2% for ranibizumab 0.3 mg, ranibizumab 0.5 mg, and sham, respectively), while progression of retinopathy was observed in only 0–4.7% of ranibizumab-injected eyes, compared with 8.7–10.5% of sham-injected eyes [19]. An interesting point that should be mentioned is that DR improvements were rapid, clinically meaningful, and sustained through month 36 [19].

Similar results were noted in a sub-analysis of the Diabetic Retinopathy Clinical Research network (DRCR.net) Protocol I, including patients with DME treated with ranibizumab. In patients with NPDR at baseline, there was ≥2-step DRSS improvement in 29%, 28% and 32% of eyes at year 1, 2, and 5 respectively. In patients with PDR, improvement was noticed in 38%, 35% and 23% of eyes at year 1, 2, and 5 respectively, concomitant with sequential reduction of anti-VEGF treatment over time [32]. Accordingly, a sub-analysis of the DRCR.net Protocol T showed that 38% of eyes with NPDR receiving intravitreal ranibizumab had DR improvement at 1 year, which remained at year 2 (31%). In the subset of patients with PDR at baseline, the 1-year DR improvement rate for patients receiving ranibizumab was 55% and maintained at year 2 despite the reduced number of injections in the second year [31].

The landmark clinical trial, examining the clinical efficacy of ranibizumab in patients with PDR was the DRCR.net Protocol S, which was a multicenter, randomized, non-inferiority phase III study, comparing intravitreal ranibizumab 0.5 mg with PRP in patients with PDR with or without DME at baseline [22,30]. Protocol S showed that ranibizumab was non-inferior to PRP in visual acuity improvement at year 2 and year 5 of follow-up [22,30]. At year 2, 35% of eyes in the ranibizumab group and 30% in the PRP group were found to have inactive or regressed neovascularization at the disc or elsewhere on fundus photographs, not differing between the two groups [30]. However, note that at year 2, a ≥2-step improvement in the DR severity score (DRSS) from baseline was observed in 42.3% of eyes treated with ranibizumab and in 23.1% of eyes treated with PRP, showing statistically significant difference between the two groups in favor of ranibizumab [30]. Note that in cost-effectiveness analyses, 0.5 mg ranibizumab was cited as cost-effective for eyes presenting with PDR and vision-impairing DME, but not for those with PDR without DME [44,45].

### 3.2. Aflibercept in the Treatment of Diabetic Retinopathy

Aflibercept is composed of the extracellular binding domains from human VEGFR1 and VEGFR2 fused to the F_c_ segment of human immunoglobulin-G1 backbone. Similar to ranibizumab, aflibercept binds to all isomers of the VEGF-A family, while it also binds to VEGF-B and placental growth factor [46,47,48]. Of note, aflibercept can regulate retinal inflammation elicited by high glucose through blocking placental growth factor signaling and seems to have protective effects on retinal cells by inhibition of the extracellular signal regulated kinases’ pathway, which may be useful in the management of early phases of DR when the inflammatory process is largely involved [47]. In addition, the affinity of aflibercept to VEGF is much higher (~140 times higher) than ranibizumab and the molecule’s intermediate size of 115 kDa (compared to 48 kDa for ranibizumab) creates a one-month intravitreal binding activity that exceeds ranibizumab [48,49,50]. Moreover, in silico data of anti-VEGF/VEGFA complexes have shown that ranibizumab and aflibercept are considerably different both in terms of molecular interactions and stabilizing energy [51].

Intravitreal aflibercept 2 mg/0.05 mL (Eylea, Bayer Healthcare Pharmaceuticals, Berlin, Germany) has been recently approved by FDA for the treatment of DR [52,53], based on the results of VIVID and VISTA, as well as PANORAMA (“A Phase 3, Double-Masked, Randomized Study of the Efficacy and Safety of Intravitreal Aflibercept Injection in Patients With Moderately Severe to Severe Nonproliferative Diabetic Retinopathy”) studies [17,34,54,55]. The VIVID and VISTA trials were two multicenter, double-masked, controlled studies including a total of 872 patients with DME, who were randomized as 1:1:1 to receive either aflibercept 2 mg every 8 weeks following five initial monthly injections, or aflibercept 2 mg every 4 weeks, or macular laser photocoagulation at baseline and then as needed. In these studies, statistically significant improvement in visual acuity was noticed in both aflibercept treatment regimens compared to the control group, which maintained at week 100 and week 148 in both studies [17,54,55]. Note that treatment with aflibercept had also positive effects on DR; greater proportion of eyes treated with aflibercept (either 2q4 or 2q8) versus those treated with laser control had a significant improvement of ≥2 steps in DRSS score in both VISTA (29.9% and 34.4% vs. 20.1% for aflibercept 2q4, aflibercept 2q8 and control, respectively) and VIVID trials (44.3% and 47.8% vs. 17.4% for aflibercept 2q4, aflibercept 2q8 and control, respectively) at week 148 [17]. More specifically, in a sub-analysis of VIVID and VISTA trials among patients with an assessable baseline DRSS score, most showed moderately severe of severe NPDR [20]. In those patients, at week 100, 29.3%, 32.6%, and 8.2% of patients treated with aflibercept 2q4, 2q8, and laser, respectively, had a ≥2 step improvement in DRSS score in VIVID and 37.0%, 37.1%, and 15.6%, respectively, in VISTA, while the proportions with ≥3 step improvement in DRSS score were 7.3%, 2.3%, and 0%, respectively, in VIVID, and 22.7%, 19.9%, and 5.2%, respectively, in the VISTA study [20]. Additionally, the progression to PDR at week 100 was less for patients treated with aflibercept compared to those in the laser group (3.2% and 2.0% vs. 12.3% for aflibercept 2q4, 2q8, and control group, respectively, in VIVID and 1.5% and 2.2% vs. 5.3% in VISTA study) [20].

Accordingly, a sub-analysis of the DRCR.net Protocol T showed that about 31% of eyes with NPDR receiving intravitreal aflibercept had DR improvement at 1 year, which remained at year 2 (~25%). In the subset of patients with PDR at baseline, the 1-year DR improvement rate for patients receiving aflibercept was 75.9% and maintained at year 2 despite the reduced number of injections in the second year [31]. Of note, in NPDR patients, there was no statistically significant difference in DRSS improvement between ranibizumab and aflibercept, but aflibercept was found superior in PDR patients compared to ranibizumab [31].

Another pivotal trial regarding the efficacy of intravitreal aflibercept in DR was the PANORAMA study, which compared intravitreal aflibercept versus sham in patients with moderately severe to severe NPDR without DME [34]. In this study, patients were randomly assigned in a 1:1:1 ratio to either three monthly aflibercept 2 mg injections followed by one injection after 8 weeks and then one injection every 16 weeks (16-week regimen); or 5 monthly aflibercept 2 mg injections followed by one injection every 8 weeks (8-week regimen); or sham treatment. The results showed that at week 52, 65% and 80% of eyes treated with 16-week and 8-week aflibercept, respectively, versus 15% of sham eyes had a ≥2-step improvement in DRSS score, which differed significantly between aflibercept treated eyes and sham treated eyes. At week 100, the same level was achieved by 62% and 50% of 16-week and 8-week aflibercept eyes respectively, versus 13% of sham eyes. It is worthy to mention that through week 52, 4% of 16-week aflibercept eyes and 3% of 8-week aflibercept eyes versus 20% of sham eyes developed a vision-threatening complication, while intravitreal aflibercept reduced the risk of developing a vision-threatening complication by 85% and 88% compared to sham (16-week and 8-week groups, respectively). However, at week 100, patients treated with intravitreal aflibercept had a 75% to 79% reduction in likelihood of developing a vision-threatening complication or DME as compared with patients in the sham arm, which could be attributed to the less frequent dosing during year 2. No safety signals were identified with aflibercept treatment over the first year of the study, suggesting that intravitreal aflibercept improved DR and prevented disease progression in eyes with moderately severe to severe NPDR without DME [34].

The recently published PROTOCOL W (“Anti-VEGF Treatment for Prevention of PDR/DME”) has examined the efficacy of intravitreal 2 mg aflibercept injections compared with sham treatment in preventing potentially vision-threatening complications in eyes with moderate to severe NPDR without DME, following a protocol of injections at baseline, 1, 2, and 4 months after baseline and every 4 months through 2 years, while between 2 and 4 years, treatment was deferred if the eye had mild NPDR or better. In addition, aflibercept could be administered in both groups if DME with vision loss or high-risk PDR developed. The results of the study showed that the proportion of eyes that exhibited PDR or vision-reducing DME was lower with periodic aflibercept compared to sham treatment. However, preventive treatment did not confer visual acuity benefit at the 2-year follow-up [36].

As far as PDR is concerned, the RECOVERY study (“Intravitreal Aflibercept for Retinal Non-Perfusion in Proliferative Diabetic Retinopathy”) evaluated prospectively the effect of intravitreal 2 mg aflibercept either monthly or quarterly in DRSS and visual function in patients with PDR without DME over a period of 12 months [35]. The study demonstrated that there was a statistically significant regression in DRSS from baseline to month 12 in both groups, which was associated with improvement in the mean composite score of visual function questionnaires (VFQ)-25 and VFQ-39, while no difference was found between the two groups [35].

The CLARITY study (“Clinical efficacy of intravitreal aflibercept versus panretinal photocoagulation for best corrected visual acuity in patients with proliferative diabetic retinopathy at 52 weeks: a multicenter, single-blinded, randomized, controlled, phase 2b, non-inferiority trial”) was a landmark, phase 2b, clinical trial regarding the efficacy of intravitreal aflibercept compared to the gold standard PDP in patients with PDR. The results of the study, including 232 patients with PDR, showed that aflibercept was not only non-inferior but also superior to PRP in terms of visual acuity improvement at year 1 of the follow-up without safety concerns [23]. New-onset center involved DME (29% vs. 11%), vitreous hemorrhage (18% vs. 9%), need for vitrectomy (6% vs. 1%), and visual loss (10% vs. 5%) were more likely to occur in eyes treated with PRP than with aflibercept [23]. In addition, intravitreal aflibercept achieved an earlier and complete regression of neovascularization in PDR compared with PRP, although there were no significant differences in global change in intravascular oxygen saturation or areas of retinal nonperfusion between the two groups by 52 weeks [56].

### 3.3. Combination of Intravitreal Anti-VEGF Agents and Panretinal Photocoagulation in the Treatment of Proliferative Diabetic Retinopathy

Several studies pointed out to the combination of intravitreal anti-VEGF agents with PRP for the treatment of PDR. Filho et al. compared intravitreal 0.5 mg ranibizumab with PRP versus PRP alone for the treatment of high-risk PDR in 40 patients [57]. They found significant reduction in fluorescein angiography leakage in both groups through week 48, but the reduction was significantly greater in the combination group, along with significant improvement in visual acuity and central retinal thickness [57].

Similar results were reported in the prospective, randomized PROTEUS study (“Prospective, Randomized, Multicenter, Open-label, Phase II/III Study to Assess Efficacy and Safety of Ranibizumab 0.5 mg Intravitreal Injections Plus Panretinal Photocoagulation (PRP) Versus PRP in Monotherapy in the Treatment of Subjects With High Risk Proliferative Diabetic Retinopathy”), in which intravitreal 0.5 mg ranibizumab plus PRP was compared to PRP alone in the regression of neovascularization area in patients with high-risk PDR without DME over a period of 12 months. The PROTEUS study showed that at month 12, 92.7% of participants in the combination group presented total reduction of neovascularization versus 70.5% of the PRP monotherapy participants, which differed significantly in favor of the combination group. Complete regression of neovascularization was observed in 43.9% in the combination group versus 25.0% in the PRP monotherapy group, which also differed in favor of combination group, although there was no difference in visual acuity change at month 12 between the two groups [24].

Furthermore, the PRIDE study (“Multicenter 12 Months Clinical Study to Evaluate Efficacy and Safety of Ranibizumab Alone or in Combination With Laser Photocoagulation vs. Laser Photocoagulation Alone in Proliferative Diabetic Retinopathy”) compared intravitreal 0.5 mg ranibizumab alone, PRP alone, and combination of them in 106 patients with PDR and no DME, showing that at month 12, there was a statistically significant greater improvement in visual acuity in the combination group, which was consistent with the stronger effect of the ranibizumab either alone or in combination with PRP on neovascularization leakage and area reduction [33]. This was in line with Chatziralli et al., who demonstrated that both intravitreal ranibizumab 0.5 mg alone and in combination with PRP could be used effectively for the treatment of PDR and co-existent DME, although the combination group presented greater regression of neovascularization with less injections [58]. Accordingly, Ferraz et al. concluded that the combination of intravitreal ranibizumab and PRP can be an effective treatment in eyes with non-high-risk PDR and DME [59]. Note that in cases where combination of anti-VEGF and PRP is used, Cao et al. have observed that the sequence of intravitreal ranibizumab before PRP showed clear advantages over that in PRP before intravitreal injection, not only in the use of lower energy for PRP, but also in the more rapid regression of neovascularization and less need of additional treatment [60]. In the long-term follow-up, however, the two-year results of the above-mentioned PRIDE study showed that discontinuation of ranibizumab treatment in PDR patients may results in an increase of neovascularization area and visual loss, suggesting that tight monitoring of disease activity and continued treatment beyond the first year are needed to maintain disease control [61].

### 3.4. Intravitreal Anti-VEGF Agents in the Management of Vitreous Hemorrhage Due to Proliferative Diabetic Retinopathy

Vitreous hemorrhage is a devastating complication of PDR, leading to significant vision loss [5]. Pars plana vitrectomy was considered the treatment of choice for eyes in which VH is not resolving. Furthermore, during PPV, visible vitreoretinal traction is generally removed along with vitreous scaffolding, preventing subsequent TRD [62,63]. The Diabetic Retinopathy Vitrectomy Study (DRVS) supported that there is a benefit of early vitrectomy in patients with type 1 diabetes, exhibiting severe VH, while the majority (80%) of patients with type 2 diabetes and severe VH still require a vitrectomy to resolve the VH after 1 year [64]. The results of PPV with current techniques seem to be better than in DRVS. Studies published within the last decade showed an improvement in visual outcome when compared with previously reported outcomes from the DRVS, with significantly less complications [65]. Therefore, given the reduced systemic risk of surgery, improved visual and anatomic outcomes of PPV with current techniques, and the reduced post-operative discomfort and recovery time, surgeons have been performing PPV for diabetic VH earlier than the 3 months that had once been the widely adopted standard for observation [63].

Intravitreal anti-VEGF agents have been evaluated as pretreatment on the outcome of vitrectomy surgery for advanced PDR. In a pilot randomized double-masked study, intravitreal ranibizumab or saline (control) was used 7 days prior to PPV in patients with TRD associated with PDR. At month 3, there was no difference in the progression of TRD prior to surgery, the duration of surgery, or its technical difficulty between the two groups, although ranibizumab reduced the extent of post-operative vitreous cavity hemorrhage, while the study was underpowered to reveal a difference in visual outcome [64].

The rationale of the use of intravitreal anti-VEGF agents for the treatment of VH before vitrectomy for patients with complicated PDR pertains to the fact that VEGF is a key molecule, which is involved in the pathogenesis of PDR, leading to neovascularization formation. Therefore, it could be hypothesized that anti-VEGF agents might facilitate much easier surgery and better visual rehabilitation, help in neovascularization regression, reduce the rate of early recurrent VH and accelerate its absorption, especially in cases of recurrent VH or residual VH post-PPV [65,66,67,68]. However, one should take into account that TRD may occur in 10% of eyes after anti-VEGF injection with the main risk factors to be the days between anti-VEGF injection and vitrectomy, VH, and age [69].

The DRCR.net Protocol N was a phase III, double-masked, randomized study, including 261 patients with VH from PDR precluding PRP, who received either intravitreal 0.5 mg ranibizumab or intravitreal saline at baseline, as well as at weeks 4 and 8 after baseline. The study showed no difference between ranibizumab and saline on the rate of vitrectomy by 16 weeks in eyes with VH from PDR, although the mean visual acuity improvement from baseline to 12 weeks was significantly greater in ranibizumab group, accompanied with lower recurrent VH within 16 weeks in the ranibizumab group [70].

Accordingly, Chelala et al. investigated in a prospective study the efficacy of intravitreal ranibizumab injections in PDR associated with VH. The authors graded VH into mild, moderate, and severe, and randomized patients into those treated with intravitreal injections of ranibizumab and those assigned to observation alone, who served as control group. Both groups could undergo PPV in the absence of improvement by 16 weeks or if there was any aggravation of the VH. The study showed that significantly better visual acuity measurements were recorded on all follow-up visits in the ranibizumab group. Moreover, there was a statistically significant difference in the vitrectomy rate in favor of ranibizumab in patients with mild-to-moderate VH, but no change in the overall vitrectomy rate and in the vitrectomy rate in severe VH, suggesting that intravitreal ranibizumab injections could be considered in PDR patients with mild and moderate VH [71].

Comparing intravitreal anti-VEGF with the gold standard PPV, the DRCR.net conducted a randomized clinical trial including 205 patients with vision loss due to VH from PDR, Protocol AB. In this study, initial treatment with 4-monthly intravitreal aflibercept injections was compared to PPV with PRP. The results showed that there was no statistically significant difference in the mean visual acuity letter score over 24 weeks through year 2 between the two groups. However, recurrent VH occurred at least once in 49% of patients in the aflibercept and in 15% in the vitrectomy group. Therefore, the authors underlined that the study may have been underpowered to detect a clinically important benefit in favor of initial PPV with PRP [72]. It has to be mentioned that although visual outcomes were not significantly different between treatment groups from 12 weeks through 2 years, additional findings from this study may help clinicians guide therapeutic decisions for individuals with VH. Specifically, PPV provides faster restoration of vision, reduced likelihood of recurrent VH, and greater resolution of neovascularization. In contrast, the aflibercept group experienced less frequent center-involved DME and avoided PPV in two-thirds of participants. As a result, the decision to initiate treatment using anti-VEGF injections versus PPV with PRP may be affected by several factors, such as the adherence of patients, medical comorbidities, and the need or desire to hasten visual recovery, particularly for patients whose fellow eye also does not have good vision [72].

### 3.5. Safety and Tolerability of Intravitreal Anti-VEGF Agents in Patients with Diabetic Retinopathy

Based on the major clinical trials evaluating the use of intravitreal anti-VEGF agents for DR, including RISE/RIDE studies, VIVID/VISTA studies, DRCR.net Protocols I, T and S, anti-VEGF agents are generally well tolerated with a good overall safety profile [19,20,30,31,32,42,43]. The majority of reported adverse reactions are related to the intravitreal injection procedure. The most frequently ocular adverse reactions are trivial, such as eye pain, ocular hyperemia, foreign body sensation, increased lacrimation, subconjunctival hemorrhage, transient increased intraocular pressure, vitreous floaters, and vitreous detachment. Less frequently reported, but more serious side-effects include endophthalmitis, retinal tear, retinal detachment, iatrogenic traumatic cataract and blindness [19,20,30,31,32,42,43].

Systemic adverse events of intravitreal anti-VEGF agents are also a concern. In RIDE/RISE study, the incidence of systemic events was overall similar between ranibizumab and control groups, and although rates of death and cerebrovascular accident were numerically higher among ranibizumab treated patients in RIDE/RISE, these were not observed in Protocol S or other ranibizumab trials [30,43]. Specifically, the systemic events seem to be dose-dependent, as the RISE/RIDE studies found an increase in incidence of stroke in ranibizumab 0.5 mg, compared with 0.3 mg dose (4.8 vs. 2.0%, respectively), while the incidence of myocardial infarction at 36 months was 7.2% in the 0.3 mg cohort and 3.6% in the 0.5 mg cohort, although the study was not powered to detect a difference in myocardial infarction incidence between the cohorts [42,43]. In VIVID/VISTA study, the overall non-ocular side effects were similar between the three groups and related to underlying comorbidities [55]. No difference was found among anti-VEGF agents in Protocol T [31], while Protocol S also did not find a difference in serious adverse events between PRP and ranibizumab [30], supporting that intravitreal anti-VEGF agents are a safe treatment alternative in patients with DR, given that this subset of patients has comorbidities and may be prone to cardiovascular events.

### 3.6. Challenges in the Use of Intravitreal Anti-VEGF Agents in Diabetic Retinopathy

Although intravitreal anti-VEGF agents have been found to be effective for the treatment of DR, providing improvement in NPDR progression and regression of neovascularization in PDR, there are some issues that should be taken into account, when treating a patient with DR using intravitreal anti-VEGF agents.

First, patients of clinical trials are inherently different from patients in real-world settings [73]. Many patients with PDR are active, working adults, whereas others have multiple comorbidities (e.g., nephropathy and cardiovascular disease) that need specialized care and treatment [74]. Patients with DR, especially PDR, often miss their scheduled medical appointments or interrupt their treatment for several reasons, such as illness, non-compliance and financial issues. Loss to follow-up in patients with PDR has been associated with younger age, lower income, race, and treatment procedure with PRP to have higher percentage of patients who are lost to follow-up (28% vs. 22.1% in patients treated with intravitreal anti-VEGF agents) [75]. Noticeably, loss to follow-up may contribute to vision loss in patients with active PDR. Recent studies have reported the risk of poor adherence in PDR, especially in eyes treated with anti-VEGF agents. Obeid et al. examined retrospectively the anatomical and functional outcomes in eyes with PDR that were lost to follow-up for more than 6 months after treatment with either intravitreal anti-VEGF or PRP. In both groups, there was a significant worsening in visual acuity at the return visit compared to the visit before being lost to follow-up, although finally the PRP group exhibited better functional outcomes at the end of the follow-up period. However, in patients with PDR treated with intravitreal anti-VEGF agents, there was a significantly greater number of eyes with TRD, as well as greater incidence of iris neovascularization, compared with the PRP group [76]. As eyes with PDR that received only intravitreal anti-VEGF agents exhibited worse anatomic and functional outcomes when being lost to follow-up compared with eyes that received PRP, the choice of treatment for PDR should be carefully considered [75,76,77,78].

Another interesting point that should be taken into account pertains to the fact that the above-mentioned clinical trials have assessed the DR improvement, mainly based on color fundus photographs. Recent studies, using newest imaging modalities, such as wide-field fluorescein angiography and optical coherence tomography angiography (OCTA) have shown conflicting results. Most of studies reported improvement in DRSS in patients treated with intravitreal anti-VEGF agents with regression of neovascularization in PDR and improvement in NPDR signs on fundus photography [79,80,81,82,83]. However, the non-perfusion areas in both macula and periphery were found to be stable, suggesting that intravitreal anti-VEGF may not reverse subsequent retinal ischemia [79,80,81,82,83,84,85]. On the other hand, animal studies have shown possible harmful cellular effects following VEGF inhibition, raising concern for potential risks of anti-VEGF treatment in patients with retinal ischemia [84,85,86]. In fact, some authors found worsening of macular ischemia with enlargement of foveal avascular zone (FAZ) area after anti-VEGF treatment [87,88,89]. Furthermore, there is controversy in the existing literature regarding peripheral ischemia in DR patients after treatment, mainly dependent on the imaging modality used for assessing non-perfusion. In studies, using ultra-widefield-OCTA, there was no progression of retinal non-perfusion or improvement in retinal perfusion in the periphery [79,90,91,92]. However, more recent reports based on swept source-widefield OCTA showed no re-perfusion in patients with DR undergoing intravitreal anti-VEGF injections [93]. Potential mechanisms explaining reperfusion may entail the restoration of the normal retinal architecture, the remodeling of pericytes, and the normalization of the basement membranes, allowing for the retinal microvasculature to regrow [91]. In eyes that do not re-perfuse, it can be hypothesized that the ischemic areas are either irreversibly infarcted or may require a higher or more frequent dose of VEGF inhibitors [92,93,94].

Note that in comparison to other indications, such as neovascular age-related macular degeneration, retinal vein occlusion, and DME, the treatment regimen for DR treatment is not clearly defined. In the pivotal trials of anti-VEGF agents on DME, as well as in Protocol S for PDR, a loading phase of ranibizumab or aflibercept was applied [17,19,20,22,30], while subsequent intravitreal injections could be administered on a pro-re-nata (PRN) basis according to the individual clinical response. The same could apply for DR treatment, but no current evidence clearly defines the exact treatment regimen for DR, neither for loading phase nor for re-injection decision. Additionally, since the main evidence regarding the use of anti-VEGF in DR derives from post hoc analyses of previous randomized controlled trials on DME, an interesting point that should be taken into account is that these studies were not designed to evaluate the direct efficacy of ranibizumab in DR; therefore, one should be very careful while interpreting their results [27].

### 3.7. Future Implications

Diabetic retinopathy is a multifactorial disease, in which several pathways are involved [4,5]. Anti-VEGF agents primarily target one of these pathways, reducing blood vessel leakage and proliferation. The angiopoietin (Ang)-tyrosine kinase with immunoglobulin-like domains (Tie) signaling pathway has been also implicated in vascular homeostasis, controlling vessel permeability, inflammation, and angiogenic response [95]. The activation of Tie2 signaling with angiopoietin 1 (Ang-1) promotes vascular stability and inhibits vascular permeability, enhancing also pericyte recruitment [96,97,98]. Additionally, Ang-2 competitively binds to Tie2, inhibiting Ang-1 signaling and leading to vascular destabilization and disruption of the blood–retinal–barrier. As VEGF has been also involved in the above-described pathways, blocking Ang-2-Tie2 and simultaneous VEGF may provide better efficacy in patients with DR [96,97,98].

Faricimab is a novel anti-Ang2/anti-VEGF bispecific antibody, which binds both VEGF-A and Ang-2 with high affinity and specificity [98,99]. The BOULEVARD clinical trial is a phase 2 study, assessing the efficacy and safety of faricimab. The preliminary results of the study showed that the percentage of patients achieving ≥2-step improvement in DRSS from baseline to week 24 were 12.2%, 27.7%, and 38.6% in the 0.3 mg ranibizumab, 1.5 mg faricimab, and 6.0 mg faricimab, respectively, in favor of faricimab, although in patients previously treated with intravitreal anti-VEGF agents, there was no difference between ranibizumab and faricimab [98].

Another pathway that recently gained scientific interest in DR is that of kallikrein-kinin. Recent evidence suggests that kinins play a primary role in the development of DR, enhancing vascular permeability, leukocytes infiltration and other inflammatory mechanisms. Therefore, ocular inhibition of kallikreins or antagonism of kinin receptor may offer new therapeutic potential for the treatment and management of DR [100,101]. KVD001 (KalVista Pharmaceuticals) is a highly potent and selective plasma kallikrein inhibitor, currently being developed as an intravitreal therapy [102]. Safety and preliminary efficacy of intravitreally injected KVD001 (1, 3, and 10 μg/eye) have been assessed in an open-label, single ascending dose clinical study, examining patients with DME, who were poor responders to previous anti-VEGF treatment, showing promising results [103]. A phase II, sham-controlled, double-masked study, enrolled approximately 123 patients with persistent DME, previously treated with intravitreal anti-VEGF agents, assessing two doses of KVD001, 6 μg and 3 μg, injected at baseline and then monthly over 3 months with 3 additional months of follow-up. In this study, no significant change was noted on DRSS, although the study population demonstrated a protective effect against visual loss [102].

Scientific interest has been also focused on the purinergic P2X7 receptor, which has been proposed as promising pharmacological target in several ocular diseases, including DR. Specifically, pericytes were found to regulate the diameter of retinal microvessels’ lumen through purinergic P2X7R, activated by the vasoactive molecule adenosine triphosphate ATP. Sustained activation of P2X7R may lead to cell apoptosis, due to formation of a wide pore through which high molecular weight molecules permeate in the cytosol, indicating P2X7R antagonism as potential pharmacological strategy to treat DR [104].

Finally, other therapies targeting not only the vascular but also the neuronal component of DR, which can precede microvascular abnormalities, are on the pipeline, including the pathway of hypoxia-inducible factor 1α (HIF1α)–6-phosphofructo-2-kinase–fructose-2,6-bisphosphatase 3 (PFKFB3) pathway. In fact, PFKFB3 is a key to the sprouting angiogenesis along with VEGF by determining the endothelial tip-cell competition, while the PFKFB3-driven glycolysis compromises the antioxidative capacity of neurons leading to neuronal loss and reactive gliosis [105]. Future studies are needed to warrant the role of new pathways in DR and potentially examining other therapeutic targets.

## 4. Conclusions

Intravitreal anti-VEGF therapy is the standard-of-care for DME, providing improvement which seems to maintain over time with limited adverse events. Moreover, it has been shown that anti-VEGF agents may improve DR severity, although there is no general consensus and no specific protocol for anti-VEGF use in patients with DR. In fact, there is lack of evidence to help physicians determine when to discontinue injection and when to retreat in patients with NPDR. Regarding PDR, based on the existing literature, both PRP and anti-VEGF agents are viable treatment options, while specific factors, such as cost and compliance, should be considered when choosing a treatment in patients with PDR. Given the chronic nature of PDR and the pharmacokinetics of intravitreal anti-VEGF agents, one of the disadvantages of anti-VEGF monotherapy for PDR is that these drugs have to be administered periodically for some time, while interruption of treatment could be devastating and lead to irreversible visual loss. Therefore, the high “loss to follow-up” rate in patients with PDR should be taken into account in the decision of treatment, based on an individualized approach. Combination treatment of intravitreal anti-VEGF agents and PRP may be a reasonable alternative for PDR.

In addition, although improvement in DR severity is shown on fundus photographs, the use of new imaging modalities, such as ultra-widefield fluorescein angiography and widefield-optical coherence tomography angiography, reveals conflicting results about the impact of anti-VEGF agents on retinal non-perfusion in patients with DR. Note that this conclusion is mainly based on *post hoc* analyses of studies designed for DME and should be interpreted with caution. Future studies targeting multiple molecular pathways are needed to optimize the treatment of DR.

## Figures and Tables

**Table 1:** Main findings of the studies included in the review.

	Design	Number of Eyes	Treatment	Follow-Up	Main Outcomes
Gross et al. (2015) [30]	Randomized clinical trial (PROTOCOL S)	394 eyes with PDR with/without DME	Panretinal photocoagulation or Ranibizumab 0.5 mg eyes with DME have received ranibizumab	2 years	Among eyes with PDR, treatment with ranibizumab resulted in visual acuity that was noninferior to PRP treatment at 2 years (visual acuity change was +2.8 in the ranibizumab group vs. +0.2 in the PRP group, *p* < 0.001). Mean peripheral visual field sensitivity loss was worse (*p* < 0.001), vitrectomy was more frequent (*p* < 0.001), and DME development was more frequent (28% vs. 9%; *p* < 0.001) in the PRP group vs. the ranibizumab group, respectively.
Bressler et al. (2017) [31]	Secondary analysis of PROTOCOL T	650 eyes with DME	Aflibercept 2.0 mg or ranibizumab 0.3 mg or bevacizumab 1.25 mg (every 4 weeks through 2 years following a re-treatment protocol)	2 years	At 1 and 2 years, eyes with NPDR receiving anti-VEGF treatment for DME may experience improvement in DR severity. Aflibercept was associated with more improvement at 1 and 2 years in the smaller subgroup of participants with PDR at baseline. All three anti-VEGF treatments were associated with low rates of DR worsening. Specifically, at 1 year, among 423 NPDR eyes, 44 of 141 (31.2%) treated with aflibercept, 29 of 131 (22.1%) with bevacizumab, and 57 of 151 (37.7%) with ranibizumab had improvement of DR severity (*p* = 0.004 for aflibercept vs. bevacizumab; *p* = 0.01 for ranibizumab vs. bevacizumab; and *p* = 0.51 for aflibercept vs. ranibizumab). At 2 years, 33 eyes (24.8%) in the aflibercept group, 25 eyes (22.1%) in the bevacizumab group, and 40 eyes (31.0%) in the ranibizumab group had DR improvement; no treatment group differences were identified. For 93 eyes with PDR at baseline, 1-year improvement rates were 75.9% for aflibercept, 31.4% for bevacizumab, and 55.2% for ranibizumab (*p* < 0.001 for aflibercept vs. bevacizumab; *p* = 0.09 for ranibizumab vs. bevacizumab; and *p* = 0.02 for aflibercept vs. ranibizumab). These rates and treatment group differences appeared to be maintained at 2 years.
Sivaprasad et al. (2017) [23]	Phase 2b, non-inferiority trial CLARITY study	232 eyes with PDR	Aflibercept 2.0 mg or PRP	1 year	Aflibercept was non-inferior and superior to PRP in both the modified intention-to-treat population mean best corrected visual acuity difference 3.9 letters (*p* < 0·0001) and the per-protocol population (4·0 letters, *p* < 0·0001). New-onset center involved DME (29% vs. 11%), vitreous hemorrhage (18% vs. 9%), need for vitrectomy (6% vs. 1%), and visual loss (10% vs. 5%) were more likely to occur in eyes treated with PRP than with aflibercept.
Wykoff et al. (2018) [19]	Post hoc analysis of RISE and RIDE	746 eyes with DME	Ranibizumab 0.3 mg or ranibizumab 0.5 mg or sham	36 months	Ranibizumab treatment resulted in DR improvements in all 3 baseline DR severity subsets examined. The greatest benefits in DR improvement occurred in patients with baseline moderately severe to severe NPDR (DR levels 47/53). Specifically, in patients with baseline DR levels 47/53, ranibizumab treatment reduced the probability of patients experiencing a new proliferative event at month 36 by 3 times compared with sham treatment (12.4% and 11.9% vs. 35.2% for ranibizumab 0.3 mg, ranibizumab 0.5 mg, and sham, respectively).
Bressler et al. (2018) [32]	Sub-analysis of PROTOCOL I	346 eyes with DME	Ranibizumab 0.5 mg	5 years	Individuals receiving ranibizumab therapy for DME may have favorable changes in DR severity throughout a 5-year period concomitant with sequential reduction in anti-VEGF therapy. Among 235 participants with NPDR at baseline, there were 29%, 28%, and 32% eyes with retinopathy improvement at 1, 3, and 5 years, respectively. Among 111 participants with PDR, corresponding improvement percentages were 38%, 35%, and 23%.
Mitchell et al. (2018) [20]	Secondary and exploratory analysis of VIVID and VISTA	403 eyes (VIVID) and 459 eyes (VISTA) with DME	Aflibercept 2.0 mg every 4 weeks (2q4) or every 8 weeks (2q8) after a loading phase of 5 monthly injections or laser and sham injections	2 years	The proportions of patients treated with 2q4, 2q8, and laser with a 2-step or more improvement in DRSS score at week 100 were 29.3%, 32.6%, and 8.2%, respectively, in VIVID-DME and 37.0%, 37.1%, and 15.6%, respectively, in VISTA-DME. The proportions with a 3-step or more improvement in DRSS score were 7.3%, 2.3%, and 0%, respectively, and 22.7%, 19.9%, and 5.2%, respectively. Fewer patients in aflibercept groups versus the laser group progressed to PDR at week 100.
Figueira et al. (2018) [24]	Prospective, randomized, open-label PROTEUS study	87 eyes with high-risk PDR	Ranibizumab 0.5 mg plus PRP or PRP alone	12 months	The number of participants with neovascularization of the disc or elsewhere reductions was higher in combination group (93.3% and 91.4%, respectively) versus PRP (68.8% and 73.7%, respectively). Complete neovascularization total regression was observed in 43.9% in the combination group versus 25.0% in the PRP monotherapy group (*p* = 0.066).
Lang et al. (2019) [33]	PRIDE study	106 eyes with PDR without DME	Ranibizumab 0.5 mg or PRP or Ranibizumab 0.5 mg plus PRP	12 months	At Month 12, there was a statistically significant difference of −2.83 mm² in the least square mean change in neovascularization area between the ranibizumab monotherapy and PRP group, favoring ranibizumab (*p* = 0.0344). Visual acuity change was greater in the ranibizumab group compared with the PRP monotherapy group at Month 12 (*p* = 0.0495).
Lim (2021) [34]	PANORAMA study	Moderately severe to severe NPDR without DME	Aflibercept 2.0 mg or sham	2 years	At week 52, 65%, and 80% of eyes treated with 16-week and 8-week aflibercept, respectively, versus 15% of sham eyes had a ≥2-step improvement in DRSS score. At week 100, the same level was achieved by 62% and 50% of 16-week and 8-week aflibercept eyes respectively, versus 13% of sham eyes. At week 100, patients treated with intravitreal aflibercept had a 75% to 79% reduction in likelihood of developing a vision-threatening complication or DME as compared with patients in the sham arm.
Alagorie et al. (2021) [35]	Prospective, multicenter trial RECOVERY study	40 eyes with PDR and no DME	Aflibercept 2.0 mg monthly or quarterly	12 months	Both monthly and quarterly groups demonstrated a statistically significant regression in DRSS from baseline to month 12 (*p* < 0.001). The monthly group demonstrated a statistically significant greater regression of DRSS score at the month 6 visit compared with the quarterly group (*p* = 0.019). However, the difference between the two groups became statistically insignificant at month 12 (*p* = 0.309).
Maturi et al. (2021) [36]	Randomized clinical trial (PROTOCOL W)	399 eyes with moderate-to-severe NPDR without DME	Aflibercept 2.0 mg or sham (baseline;1,2,4 months; every 4 months through year 2)	24 months	The 2-year cumulative probability of developing PDR was 13.5% in the aflibercept group versus 33.2% in the sham group (*p* < 0.001), and the 2-year cumulative probability of developing DME with vision loss was 4.1% in the aflibercept group versus 14.8% in the sham group (*p* < 0.001). The mean change in visual acuity from baseline to 2 years was −0.9 letters with aflibercept and −2.0 letters with sham, not reaching statistical significance.

DME: diabetic macular edema; DRSS: diabetic retinopathy severity score; NPDR: non-proliferative diabetic retinopathy; PDR: proliferative diabetic retinopathy; PRP: panretinal photocoagulation.

## Data Availability

Data is contained within the article.
